# Nonreference Image Quality Evaluation Algorithm Based on Wavelet Convolutional Neural Network and Information Entropy

**DOI:** 10.3390/e21111070

**Published:** 2019-10-31

**Authors:** Jinhua Liu, Mulian Xu, Xinye Xu, Yuanyuan Huang

**Affiliations:** 1 School of Mathematics and Computer Science, Shangrao Normal University, Shangrao 334001, China; mulianml@tom.com (M.X.); sruxxy@163.com (X.X.); 2 Department of Network Engineering, Chengdu University of Information Technology, Chengdu 610225, China; iyyhuang@hotmail.com

**Keywords:** nonreference image quality evaluation, convolutional neural network, deep learning, wavelet transform, information entropy

## Abstract

The image quality evaluation method, based on the convolutional neural network (CNN), achieved good evaluation performance. However, this method can easily lead the visual quality of image sub-blocks to change with the spatial position after the image is processed by various distortions. Consequently, the visual quality of the entire image is difficult to reflect objectively. On this basis, this study combines wavelet transform and CNN method to propose an image quality evaluation method based on wavelet CNN. The low-frequency, horizontal, vertical, and diagonal sub-band images decomposed by wavelet transform are selected as the inputs of convolution neural network. The feature information in multiple directions is extracted by convolution neural network. Then, the information entropy of each sub-band image is calculated and used as the weight of each sub-band image quality. Finally, the quality evaluation values of four sub-band images are weighted and fused to obtain the visual quality values of the entire image. Experimental results show that the proposed method gains advantage from the global and local information of the image, thereby further improving its effectiveness and generalization.

## 1. Introduction

Image quality evaluation has a wide range of applications in image compression, image restoration, and video processing. Existing image quality evaluation methods mainly include subjective and objective types [1,2]. Subjective evaluation method directly evaluates or scores the image quality on the basis of people’s relevant experiences. The commonly used rating grades are excellent, good, medium, poor, and very poor. The subjective evaluation method is simple and has high accuracy. However, it needs to rely on people’s subjective experiences, and the labor costs are high. As such, this method is difficult to promote in practical applications, especially real-time processing.

Although the performance of the objective evaluation method is not as good as that of the subjective evaluation method, the image quality can be automatically evaluated by establishing the image distortion evaluation model, thereby reducing labor costs. The objective evaluation method has strong real-time performance. Therefore, it has become a popular research topic in the field of image quality evaluation in recent years. In general, objective evaluation methods include full reference [3,4], reduced reference [5,6], and nonreference [7,8,9,10,11,12]. This study focuses on the nonreference quality evaluation of images, i.e, referring to the original image information in image quality evaluation is unnecessary.Generally speaking, the nonreference image quality evaluation method is also called blind image quality (BIQ) evaluation method. It does not need reference image at all, and estimates the image quality according to the features of the distorted image.

The simplest objective evaluation methods are the peak signal-to-noise ratio (PSNR) and mean square error (MSE). These methods are simple to implement with high computational efficiency and are widely used in the visual quality evaluation of image processing. However, the two methods do not consider human visual perception psychology. The evaluation results are relatively different from the subjective judgments, and the intrinsic characteristics of human visual perception are difficult to reflect.

In recent years, deep neural networks, especially convolutional neural network (CNN) technology, which achieved better results than traditional methods in image recognition, target detection, and image restoration, achieved unprecedented development in the field of image processing [13,14]. Existing image quality evaluation methods based on CNNs mainly use two CNN characteristics. One is the local receptive field, i.e, people perceive the visual content of images from partial to global. In the image, the correlation between the pixels of the nonlocal area is low, and the correlation between the pixel points of the local area is large. The other is weight sharing, i.e., the CNN obtains the local information of the image by using the same filter operator. In general, all local information in the image obtained by the filter operator is consistent. Therefore, in an actual image quality evaluation, various filter operators may be set to extract effective features, and different feature maps may be extracted by different filter operators. The general CNN framework includes an input layer and convolutional multilayer. A pooling layer between the convolutional layers is mainly used to reduce the size of the feature map and the dimensionality of data. Therefore, the CNN gradually extracts the high-level semantic features of the image by continuously stacking the convolutional and pooling layers and finally converts the feature vector into a classifier or a fully connected layer.

Several researchers introduced image quality evaluation methods based on CNN. For example, Kang et al. [15] fused convolutional and fully connected layers to design a deep neural network that predicts image quality scores. This network used an image block with a size of 32 × 32 as an input and used the quality score of the entire image to represent the quality score of the image block. Kim et al. [16] designed a two-stage deep neural network model to evaluate image quality. In the first stage, an end-to-end deep neural network model was trained and imputed as an image block with a size of 32 × 32. The score of the image block was calculated using objective quality evaluation algorithm and used as the output of the deep neural network. In the second stage, the image block of the entire image was inputted into the deep neural network obtained in the first stage, and the features corresponding to all image blocks were merged and outputted as the quality scores of the entire image. Bare et al. [17] used 32 × 32 image blocks as inputs to the CNN similar to the network framework proposed in [16] and adopted the full reference quality evaluation algorithm [18] to calculate the quality score of the image block as the output of the CNN. They constructed the image quality evaluation network framework on the basis of the residual network method [19]. The two models proposed in [16,17] have an evident defect, i.e., the quality score calculated by the objective image quality evaluation method is used to represent the subjective quality score of the image block. Although [17] used the full reference quality evaluation algorithm [18], which can accurately predict the image quality score, the score calculated by the algorithm still had a certain gap with the subjective score.

In [20], the author believed that people provide qualitative scores of images, such as very good, good, bad, and very poor. This qualitative evaluation was converted into feature vectors to design the image quality evaluation method. Kim et al. [21] proposed an end-to-end CNN model, which inputs distorted and error images. The model was used to learn the optimal weights automatically and fuse the error images for obtaining the visual quality score of the distorted images. On the basis of the image pair generation strategy, [22] proposed a deep CNN training model, which achieved good image quality evaluation performance. Bosse et al. [23] proposed full reference and nonreference image quality evaluation methods based on deep CNN. The proposed networks mainly included the feature extraction, feature fusion, and pooling layers. Ma et al. [24] proposed an end-to-end blind image quality evaluation method combined with image distortion type prediction and quality prediction and designed a multitask CNN image quality evaluation network model. [25,26,27] proposed the corresponding nonreference image quality evaluation method, which achieved good results.

The existing image quality evaluation method based on CNN uses the average value of the image sub-block to represent the quality evaluation value of the entire image. This method can detect low- and high-quality image regions and achieve good image quality evaluation results. However, the visual quality of the partial image sub-block tends to change with the spatial position. After the image is subjected to distortion processing, the quality evaluation based on the partial image sub-block has difficulty reflecting the visual quality of the entire distorted image. On the contrary, image sub-blocks with similar distortion types (e.g., blurred or smooth regions) may also have significantly different visual qualities. The main contributions of this paper are summarized as follows:

1. We present a wavelet convolution neural network for image quality assessment. The product neural network extracts the feature information in multiple image directions, thereby further improving the effectiveness and generalization of the image quality evaluation method.
2. We adopt the information entropy as the weight of quality prediction of sub-band image, and demonstrate that the distribution of information entropy is close to the image region of human visual perception. Using this strategy, the subjective and objective consistencies of the image quality evaluation can be further improved.

## 2. Information Entropy of Sub-Band Image

### 2.1. Application of the Discrete Wavelet Transform (DWT)

Wavelet transform is an effective tool to combine time domain and frequency domain. In most applications, discrete signals are used. Therefore, discrete wavelet transform (DWT) must be used instead of continuous wavelet transform. Wavelet transform can decompose the signal by band-pass filter. The result of the band filtering operation will be two different signals, one will be related to the high frequency components and the other related to the low frequency component of the original signal.

To compute the DWT of an image I(x,y) of size M×N, it must identify the wavelet scale function $W_{\varphi }$ to define the approximation coefficients and the wavelet function $W_{\psi }$ responsible for horizontal, vertical and diagonal coefficients {H,V,D} following the equations below:

(1) $$W_{\varphi }\left(j_{0},m,n\right)=\frac{1}{\sqrt{MN}}\sum_{x=0}^{M-1}\sum_{y=0}^{N-1}I\left(x,y\right)\varphi_{j_{0},m,n}\left(x,y\right)$$

(2) $$W_{\psi }^{i}\left(j_{0},m,n\right)=\frac{1}{\sqrt{MN}}\sum_{x=0}^{M-1}\sum_{y=0}^{N-1}I\left(x,y\right)\psi_{j_{0},m,n}^{i}\left(x,y\right)$$

with:

(3) $$\varphi_{j,m,n}\left(x,y\right)=\frac{1}{2^{j}}\varphi \left(\frac{x-2n}{2^{j}},\frac{y-2m}{2^{j}}\right)$$

(4) $$1\leq i\leq 3,\psi_{j,m,n}^{i}\left(x,y\right)=\frac{1}{2^{j}}\psi^{i}\left(\frac{x-2^{j}n}{2^{j}},\frac{y-2^{j}m}{2^{j}}\right),i∼\left\{H,V,D\right\}$$

where $j_{0}$ is the start resolution and the scale parameter *j* is always greater or equal to $j_{0}$. In general, we choose $j_{0}=0$ and $N=M=2^{j}$ in order that j=0,1,…,j−1 and $m,n=0,1,\ldots ,2^{j}-1$.

### 2.2. Calculation of Information Entropy

**Figure 1:**
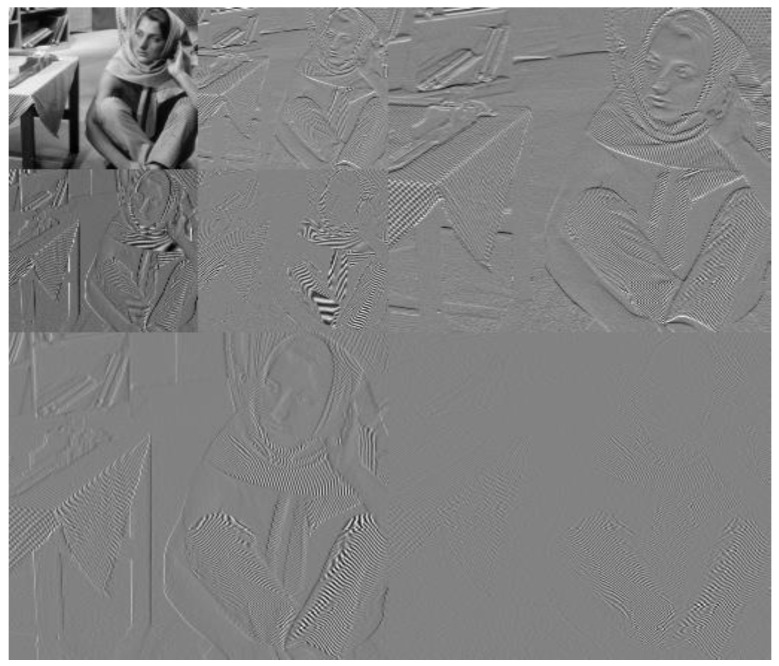
Wavelet transform of Barbara.

**Figure 2:**
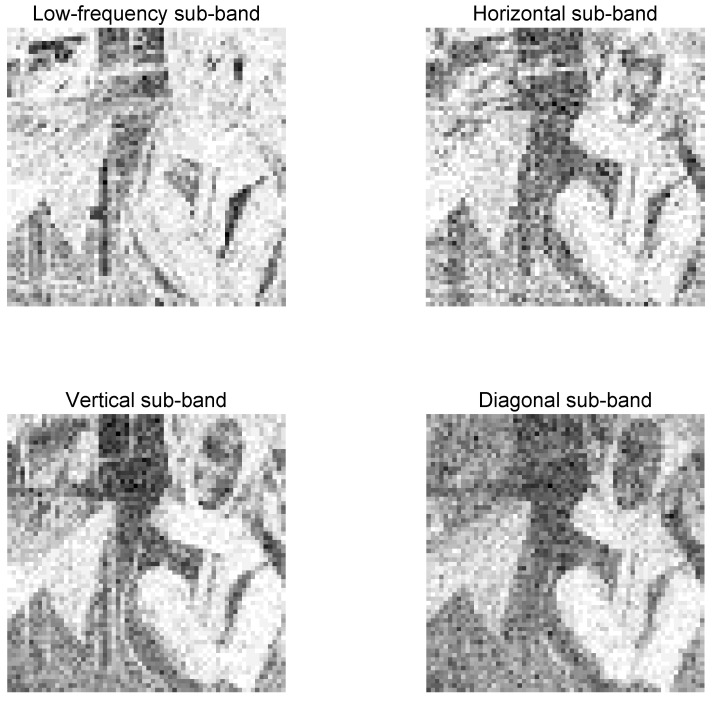
Figures of Information Entropy of different wavelet sub-bands.

After an image is transformed by wavelet [28], a series of sub-band images with different resolutions can be obtained. Figure 1 shows the results of a Barbara image with a size of 512 × 512 decomposed by two layers of wavelets. The upper leftmost part of each layer in Figure 1 is a low-frequency image, and the upper right, lower left, and upper right corners are the vertical high-frequency, horizontal high-frequency, and diagonal sub-band images, respectively. The second layer decomposes the low-frequency image of the first layer into a low-frequency sub-band image (upper left corner in Figure 2) and a high-frequency sub-band image in the vertical, horizontal, and diagonal directions. Subsequently, the third layer wavelet transform repeats this process to continue to decompose the low-frequency image of the second layer, and the like.

The above evaluation shows that the multiscale analysis of wavelet transform can efficiently describe the global and local information of the image. Generally, a low-frequency image reflects the global information of the entire image, but a high-frequency sub-band image reflects the local details, such as edge, contour, and other image areas with mutations. Therefore, this section calculates the corresponding information entropy of each wavelet sub-band image on the basis of the information of multiple directions. Then, each information entropy is used as the visual quality weight of the corresponding sub-band image to describe the effects of different sub-band images on the quality of the entire image. The calculation process of information entropy is summarized as follows:

Distorted image *I* is imput, and S-layer wavelet decomposition is performed for distorted image to obtain low-frequency, horizontal, vertical, and diagonal sub-band images, denoted as $I_{L}$, $I_{H}$, $I_{V}$, and $I_{D}$, respectively. Then, each sub-band image is divided into image sub-blocks that do not overlap, and the information entropy of each sub-block is calculated. Finally, the average information entropy of all sub-blocks is obtained and used as the visual content weight of the cost function. The number of layers S of the wavelet decomposition is set to 1. The information entropy of each sub-block is calculated as follows:

(5) $$H=-\sum_{i=1}^{N_{B}}p(w_{i})\log p(w_{i}),$$

where $p(w_{i})$ denotes the probability of wavelet coefficient $w_{i}$ appearing in the sub-block image, and $\sum_{i=1}^{n}p(w_{i})=1$. $w_{i}$ represents the wavelet coefficients of the sub-block image, $N_{B}$ is the number of all wavelet coefficients of each sub-block image.

Generally, information entropy reflects the intensity of image information to a certain extent. The larger the information entropy of an image, the larger the amount of information, and the better the visual quality of the image. Moreover, the information entropy of the image includes rich structural information, which can be used to measure the sensitivity of the local image. Therefore, people are inclined to evaluate the visual quality of images from areas with high acuity. Figure 2 presents the information entropy map of the low-frequency and three high-frequency sub-band images after the wavelet transform of Barbara image. Figure 2 also shows the large amount of structural information and the distribution of the information entropy, which is close to the image area of human visual perception. Therefore, the wavelet information entropy of the image can be used as the visual weight to improve the subjective and objective consistencies of the image quality evaluation.

## 3. Proposed Image Quality Evaluation Algorithm

**Figure 3:**
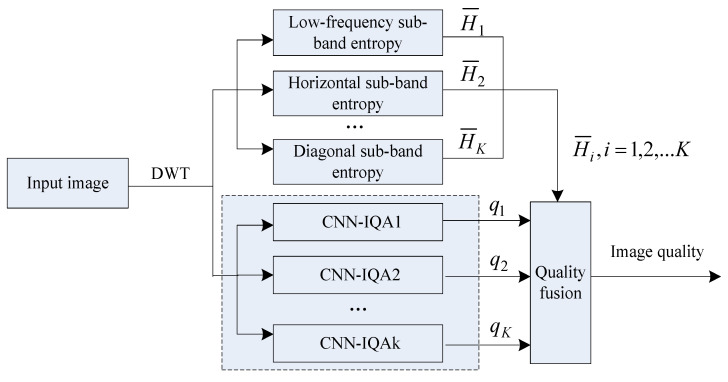
Flow chart of the proposed image quality evaluation algorithm.

To improve the robustness and generalization of image quality evaluation methods, this study combines wavelet transform and CNN to design a nonreference image quality evaluation method based on wavelet CNN. Figure 3 shows the flow chart of the proposed algorithm.

Concretely, the wavelet CNN method is used for designing the nonreference algorithm for image quality evaluation.In Figure 3, the S-wavelet transform is initially performed on the input image to be measured (the image is decomposed using a one-level wavelet transform), and the low-frequency, horizontal, vertical, and diagonal sub-band images are obtained. Because the image is decomposed by S-level wavelet, the number of sub-band images is 3S+1, i.e., the value of K is 3S+1. In our work, we decompose the image with one level wavelet transform, then we get four sub-band images, so the value of K is 4. Besides this, and “db1” is selected as the wavelet filter. These parameters do not depend on the analyzed image (such as size, texture, etc.). This is easy to handle in a unified way.

**Figure 4:**
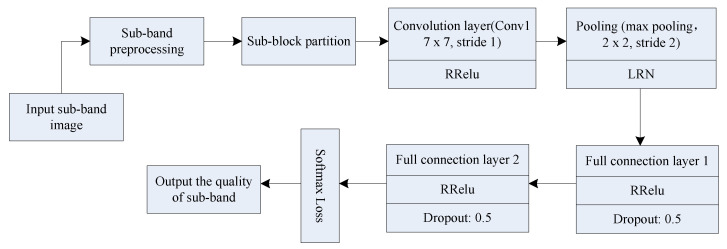
Sub-band image quality prediction based on CNN.

Next, the information entropy of each sub-band image, denoted as ${\bar{H}}_{i}$, i=1,2,3,4, is calculated. The four sub-band images are used as the inputs of the CNN to output their quality prediction values through the CNN, represented as CNN_IQA1, CNN_IQA2, CNN_IQA3, and CNN_IQA4, respectively. The quality prediction values of the four sub-band images adopt the same CNN structure. Figure 4 shows the detailed flow.

### 3.1. Image Local Contrast Normalization Preprocessing

Before using the CNN to predict the quality of the sub-band image, the image is normalized for local contrast, i.e., removing redundant features that are weakly related to image quality. The process of local contrast normalization preprocessing is as follows:

Wavelet decomposition is performed on the distorted image via wavelet transform to obtain a low-frequency sub-band image and three sub-band images in horizontal, vertical, and diagonal directions. Furthermore, to remove redundant feature information that is comprehensively weakly related to image quality, local contrast normalization preprocessing is performed on the four sub-band images. The specific process is summarized as follows:

(6) $$\tilde{I}\left(i,j\right)=\frac{I\left(i,j\right)-\mu_{I}}{\sigma_{I}+C},$$

where I(i,j) represents the initial pixel value at (i, j) in the distorted image, $\tilde{I}\left(i,j\right)$ is the normalized value at (i, j) in the distorted image, and $\mu_{I}$ and $\sigma_{I}$ are the pixel mean and standard deviation of the local area of the image, respectively. The value of constant *C* is set to 1.0. The calculation processes of $\mu_{I}$ and $\sigma_{I}$ are as follows:

(7) $$\mu_{I}=\sum_{m=-M}^{M}\sum_{m=-N}^{N}w_{m,n}I\left(i+m,j+n\right),$$

(8) $$\sigma_{I}=\sqrt{\sum_{m=-M}^{M}\sum_{m=-N}^{N}w_{m,n}{\left[I\left(i+m,j+n\right)-\mu_{I}\right]}^{2}},$$

where $w_{m,n}$ represents the weight of the Gaussian function window. The window size is set to 3 × 3, and the values of *M* and *N* are both 3.

### 3.2. Sub-Band Image Quality Prediction Based on CNN

The low-frequency sub-band image generally reflects the global information of the image, and the high-frequency sub-band image reveals the local detailed information. Therefore, the performance of the image quality evaluation method is further improved to use the global and local information of the image fully. In this section, the CNN is used to predict the low-frequency and three high-frequency sub-band images simultaneously. Initially, the method described in Section 2 of this paper is used to calculate the information entropy of each sub-band image, i.e., the information entropy of low-frequency, horizontal, vertical, and diagonal sub-band images, denoted as ${\bar{H}}_{i}$, i=1,2,3,4. The four information entropy is used as the weight of the quality prediction, and then the CNN model is trained on the four sub-band images by supervised learning. The quality prediction values of the four sub-band images adopt the same CNN.

Figure 4 shows the architecture of the proposed network, which is a 32×32−26×26×50−13×13×50−400−100−1 structure. The input is locally normalized 32 × 32 image patches of sub-band image. The first layer is a convolutional layer which filters the input with 50 kernels each size 7 × 7 with a stride of 1 coefficient. The convolutional layer produces 50 feature maps each of size 26 × 26. The obtained feature map is used as the input data of the pooling layer, then 50 feature maps with a size of 13 × 13 will be obtained. Two fully connected layers of 400 nodes and 100 nodes each come after the max pooling. The last layer is a simple linear regression with a one-dimensional output that give the quality score.

In Figure 4, the calculation process of the quality prediction value of each sub-band image is as follows:

(1) the wavelet-decomposed sub-band images, including low-frequency, horizontal, vertical and diagonal sub-band images, are inputted. Then, local contrast normalization preprocessing is performed on the four sub-band images on the basis of the method in .1.

(2) after the subband image is preprocessed, the sub-blocks are divided, assuming that the divided image subblock size is 32 × 32. A 32 × 32 subblock image is used as input data for the CNN.

(3) the CNN parameters, including convolutional, pooling, and two fully connected layers, are designed. The convolutional layer uses a convolution kernel with a size of 7 × 7, the number of convolution kernels is 50, and the sliding window step size is set to 1 in the convolution process. Then, the image subblock is convolved with the convolution kernel, and the size of the feature map activated by an activation function is (32−7)/1+1=26 pixels. Thus, 50 feature maps with a 26 × 26 size are obtained; the activation function is RReLU, which is expressed as

(9) $$y=\left\{\begin{array}{cc} x, & x\geq 0\\ ax, & x<0 \end{array}\right.$$

where *x* represents an input and *a* is a small normal number. *a* is set to 0.01 in the present study.

The obtained feature map is used as the input data of the pooling layer. The maximum pooling method is adopted in the present invention, and the step size is set to 2. Then, the feature map size obtained after the pooling is (26−2)/2+1=13 pixels. Hence, 50 feature maps with a size of 13 × 13 will be obtained. LRN represents the local response normalization process, which aims to enhance the generalization of the CNN. In Figure 4, the third and fourth layers are connected, use RReLU as the activation function, and adopt dropout processing. The purpose is to discard some of the elements in the fully connected layer from the network at a probability of 0.5 to avoid over-fitting phenomenon. Finally, the quality prediction values of each sub-band image, which are represented as $q_{1}$, $q_{2}$, $q_{3}$, and $q_{4}$, are obtained using nonlinear regression loss function Softmax loss.

In a word, the learning process is summarized as follows:

Let $p_{i}$ and *y* denote the input patch and its ground truth score respectively, $f(p_{i};w)$ refers to the predicted score of $p_{i}$ with network weights *w*. Each image patch was independently regressed onto the global subjective-quality score. The objective function can be written as

(10) $$L=\frac{1}{N}\sum_{n=1}^{N}\left\|f(p_{i};w)-y\right\|$$

where ‖.‖ denotes the $l_{1}$ norm. It is known that support vector regression (SVR) with ϵ−insensitive loss was successfully applied to learn the regression function for nonreference image quality assessment. Please note that the above loss function is equivalent to the loss function used in ϵ−SVR with ϵ=0. This problem can be solved by using the stochastic gradient descent (SGD) and back propagation (BP) method. In experiments we perform SGD for 60 epochs in training and keep the model parameters that generate the highest Pearson linear correlation coefficient (PLCC) on the test data set. The CNN model was trained via a patchwise optimization, and, during testing, the outputs of multiple patches composing an input image were averaged to obtain a final predicated subjective score.

### 3.3. Proposed Algorithm

On the basis of Section 2, Section 3.1 and Section 3.2 of this paper, the proposed algorithm steps are summarized as follows:

(1) Distorted image *I* is inputted, and a single-layer wavelet decomposition is performed on distorted image to obtain low-frequency, horizontal, vertical, and diagonal sub-band images, denoted as $I_{L}$,$I_{H}$, $I_{V}$, and $I_{D}$, respectively. On the basis of the method described in Section 2, each sub-band image is divided into image sub-blocks that do not overlap, and the information entropy of each sub-block is calculated. Finally, the average of information entropy of all sub-blocks is obtained, and the value is used as the quality prediction weight for the entire sub-band image.

(2) On the basis of the method described in Section 3.2, the images are initially normalized and preprocessed, and then the wavelet CNN model is trained. The quality prediction values of the low-frequency and three high-frequency sub-band images, denoted as $q_{1}$, $q_{2}$, $q_{3}$, and $q_{4}$, respectively, are predicted using the wavelet CNN model.

(3) Image quality fusion processing. The information entropies of the low-frequency, horizontal, vertical, and diagonal sub-band images are used as the weights of quality prediction values. Then, they are fused to obtain the quality evaluation value of the entire image. The fusion process can be expressed as follows:

(11) $$Q=\sum_{i=1}^{K}{\bar{H}}_{i}\times q_{i},K=4$$

where *Q* represents the quality prediction value of the entire image, *K* is the number of sub-band images, ${\bar{H}}_{i}$ (*i* = 1, 2, 3, 4) is the average information entropy value of the No. *i* sub-band image, and indicates the quality prediction value of the No. i sub-band image. Specifically, ${\bar{H}}_{1}$, ${\bar{H}}_{2}$, ${\bar{H}}_{3}$, ${\bar{H}}_{4}$ are the information entropies and $q_{1}$, $q_{2}$, $q_{3}$, and $q_{4}$ are the quality prediction values of the low-frequency, horizontal, vertical, and diagonal sub-band images, respectively.

(4) The test is conducted on an arbitrary image database, the image quality evaluation score is obtained by the wavelet CNN model, and the performance of the image quality evaluation method is evaluated.

The pseudocode of proposed Algorithm 1 is summarized as follows:

**Algorithm 1** NIQA algorithm based on wavelet CNN and information entropy.
**Input:** The distorted image *I*;**Output:** The predicated quality score of image *I*;1:Decompose the distorted image by one-level DWT via Equations (1) and (2); Extract four sub-band images, which denoted as $I_{L}$,$I_{H}$, $I_{V}$, and $I_{D}$;2:Divide each sub-band image into non overlapping sub blocks, and compute the information entropy of each sub-block via Equation (5),obtain the average information entropy of each sub-band image;3:Perform the local contrast normalization for these four sub-band images via Equations (6)–(8);4:Train the Wavelet CNN via the object function (10), obtain the predicated quality scores of four sub-band images, which denoted as $q_{1}$,$q_{2}$, $q_{3}$, and $q_{4}$;5:Compute the quality score of entire image via Equation (11);6:Test the wavelet CNN model on an arbitrary database to obtain the corresponding image quality prediction results.


## 4. Experimental Results and Analysis

To verify the performance of the proposed method, the experimental configuration of this study is in a Windows 10 environment, the processor is Intel Core i7-8550U, and the memory is 16 GB. The software tool is MATLAB R2018, and the deep learning library uses MatConvNet (V1.0-beta22).

### 4.1. Data Settings

On the basis of the image quality evaluation method described in this paper, we use the LIVE [29], TID2008 [30], and TID2013 [31] image databases to evaluate the proposed image quality evaluation method. The LIVE image database [29] has a total of 779 distorted images, including JPEG2000 (169), JPEG (175), Gaussian white noise (145), Gaussian blur (145), and fast fading (145). The distorted image in the LIVE image database is obtained by adding different types and levels of distortion to the 29 reference images. The TID2008 database [30] includes 25 reference images, 1700 different types and degrees of distorted images, and 17 types of distortion, including additive Gaussian noise, JPEG compression, salt and pepper noise, Gaussian blur, JPEG2000 compression, and brightness change. The subjective evaluation score of the distorted image is based on the observer’s subjective evaluation in the form of differential average subjective score. The value of difference mean opinion score (DMOS) reflects the subjective quality of the distorted image, and if the value is small, then the corresponding subjective evaluation quality is high.

The TID2013 database [31], an enhanced version of TID2008, includes 25 reference images and 3000 distorted images. A total of 24 types of distortion are observed: changing color saturation, multiple Gaussian noise, comfort noise, lossy compression, color image quantization, chromatic aberration, and sparse sampling. The DMOS value of the database is obtained by the 524,340 data provided by the 971 observers, and the mean opinion score (MOS)value range is [0, 9]. The variety of distortions in the database makes the database abundant and become a color distortion database. Therefore, numerous image quality evaluation algorithms include the database in the comparative experiment.

To analyze the image quality evaluation performance of the proposed and other methods, two indicators are used, namely, Pearson linear correlation coefficient (PLCC) and Spearman rank-order correlation coefficient (SROCC). PLCC is mainly used to evaluate the accuracy of image quality evaluation methods. The larger the value, the better the accuracy of the corresponding evaluation method. PLCC can be defined as:

(12) $$PLCC=\frac{\sum_{i=1}^{n}\left(X_{i}-\bar{X}\right)\left(Y_{i}-\bar{Y}\right)}{\sqrt{\sum_{i=1}^{n}{\left(X_{i}-\bar{X}\right)}^{2}\sum_{i=1}^{n}\left(Y_{i}-\bar{Y}\right)}}$$

where $X_{i}$ and $Y_{i}$ stand for the MOS and the model prediction of the *i*-th image, respectively. $\bar{X}$ and $\bar{Y}$ are expressed as the mean of subjective score and prediction score, respectively. *n* represents the number of test sets. SROCC mainly reflects the consistency between objective and subjective evaluations. The larger the value, the better the performance.SROCC can be defined as:

(13) $$SROCC=1-\frac{6\sum_{i}{d_{i}}^{2}}{n(n^{2}-1)}$$

where *n* is the test image number and $d_{i}$ is the rank difference between the MOS and the model prediction of the *i*-th image.

### 4.2. Results on the LIVE Database

Table 1 and Table 2 show the performance comparison results of the proposed and other methods on the LIVE database. The evaluation methods include the structural similarity methods proposed in [4], the DIIVINE evaluation method based on natural scene statistics in [10], the BRISQUE evaluation method based on spatial domain in [11], the BLIINDS-II method based on DCT in [12], the evaluation method based on CNN in [15], and the BIECON evaluation method in [16]. In the experiments corresponding to Table 1, Since the proposed image quality approach requires a training procedure to calibrate the regressor module, we divide the LIVE database into two randomly chosen subsets—80% training and 20% testing—such that no overlap between train and test content occurs.In the experiments corresponding to Table 2, the distorted images of the 23 original images in the LIVE database are selected as the training samples, and the distorted images of the 6 original images are used as the test samples for experiments. Further, to eliminate performance bias, we repeat this random train-test procedure 1000 times and report the median of the performance across these 1000 iterations.

The results in Table 1 and Table 2 show that the image quality evaluation performance of the proposed method has improved to a certain degree in most cases compared with the above mentioned evaluation methods, thereby proving its effectiveness. The reason is that this study uses the same feature of CNN and wavelet transform, i.e., multiscale analysis, which can obtain image information in multiple directions, thereby improving the generalization of image quality evaluation methods. In addition, using information entropy as the weight of visual quality evaluation can reflect the influence of different sub-band images on the visual quality of the entire image and improve the subjective and objective consistencies of image quality evaluation.

To assess the data dispersion, we added some statistical analysis work to evaluate the data dispersion of PLCC and SROCC on LIVE for the proposed image quality assessment method. The standard variance values of PLCC and SROCC were added in Table 3. From Table 3, it can be seen that the data variance of PLCC and SROCC is small, which shows that the proposed method has relatively stable performance.

**Figure 5:**
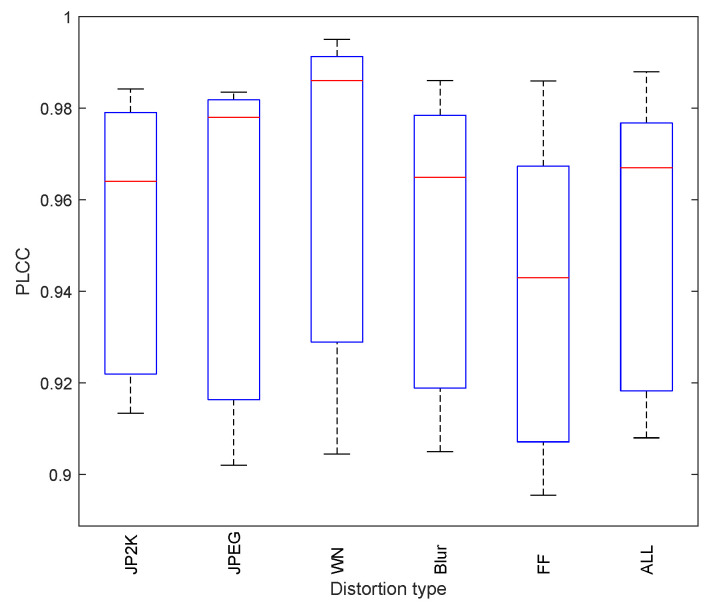
Box plot showing minimum, first quartile, media, third quartile and maximum PLCC correlation for the proposed method on LIVE dataset.

**Figure 6:**
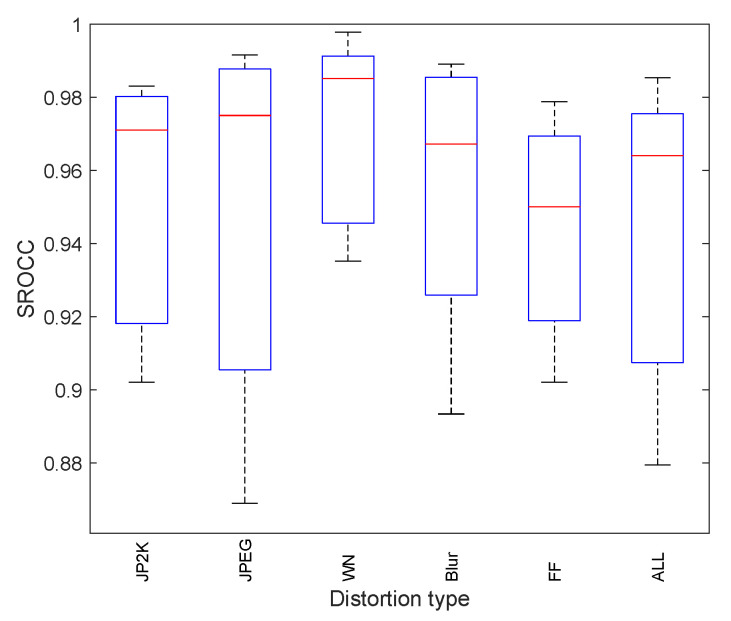
Box plot showing minimum, first quartile, media, third quartile and maximum SROCC correlation for the proposed method on LIVE dataset.

Furthermore, to evaluate the robustness of the proposed image quality algorithm on LIVE dataset, the Box plots of PLCC and SROCC correlation are showed in Figure 5 and Figure 6. It can be seen that the results obtained on the LIVE dataset demonstrate the robustness of the proposed method on different distortion types.

### 4.3. Cross-Validation on the TID2008/TID2013 Databases

To verify the adaptability of the proposed method to the new sample, this section trains the model in the LIVE image database and tests it in the TID2008 and TID2013 image databases. Since the TID2008 and TID2013 database contain more distortion types, only four types, i.e., JPEG2000 compression, JPEG, WN, and BLUR are selected in the experiment.FF distortion does not exist in the TID2008 and TID2013 database. Since the range of DMOS scores is from 0 to 100 in LIVE database, and the range of mean opinion score(MOS) scores is from 0 to 9 in TID2008. Therefore, to make a fair comparison, we adopt the same method as [32] to perform a nonlinear mapping on the predicted scores produced by the model trained on LIVE database. The detail of mapping method can be refer to literature [32]. Besides this, the TID2008 is spitted into two parts of 80% and 20% randomly. 80% of the data is randomly selected for estimating parameters of the logistic function, and 20% is used for testing, i.e., evaluating the transformed prediction scores. This random spit procedure are repeated 100 times in this work.

Table 4 and Table 5 show the results of the cross dataset test. It can be seen that the proposed image quality algorithm outperforms previous state of the art methods.Similarly, we adopt the same procedure as above to test the generalization ability of our method in TID2013 database. Furthermore, for comparison with other algorithms, we added simulation comparison results between our method and other works in dipIQ [25], CORNA [32] and ILNIQE [33] in Table 6 and Table 7. The results of the cross-dataset test are shown in the two tables. From Table 6 and Table 7, we can see that the performance of the proposed method is satisfied. Overall, the testing results on two image databases, in most cases, show that the proposed method has better performance than other image quality evaluation methods.

### 4.4. Parameter Setting and Performance Evaluation

In the wavelet CNN model proposed in this paper, some model parameters are involved, such as the size and number of convolution kernels and the proportion of the training dataset. This section specifically analyzes the effects of different settings of these parameters on network performance.

#### 4.4.1. Size of the Convolution Kernel

The network model is trained and tested using convolution kernels with different sizes without changing the network structure. Table 8 shows the comparison results of the performance with different sizes of convolution kernel networks. Convolution kernels with different sizes have a small influence on network performance. Hence, this study selects a 7 × 7 convolution kernel.

#### 4.4.2. Number of Convolution Kernels

**Figure 7:**
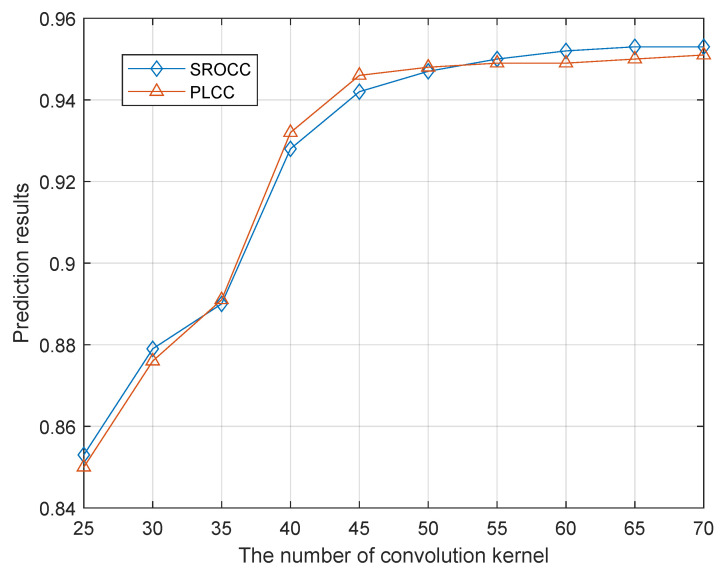
Results of quality prediction under different convolution kernels.

Figure 7 shows the relationship between the number of convolution kernels and the predicted results. The prediction result of the network increases with the increase in the number of convolution kernels. However, when the number of convolution kernels exceeds 50, the prediction result tends to stabilize; furthermore, the increasing number of convolution kernels dramatically increases the time costs required for network training. Therefore, considering the accuracy and time efficiency of network prediction, the number of convolution kernels is set to 50.

#### 4.4.3. Analysis of Prediction Results with Different Training Set Sizes

**Figure 8:**
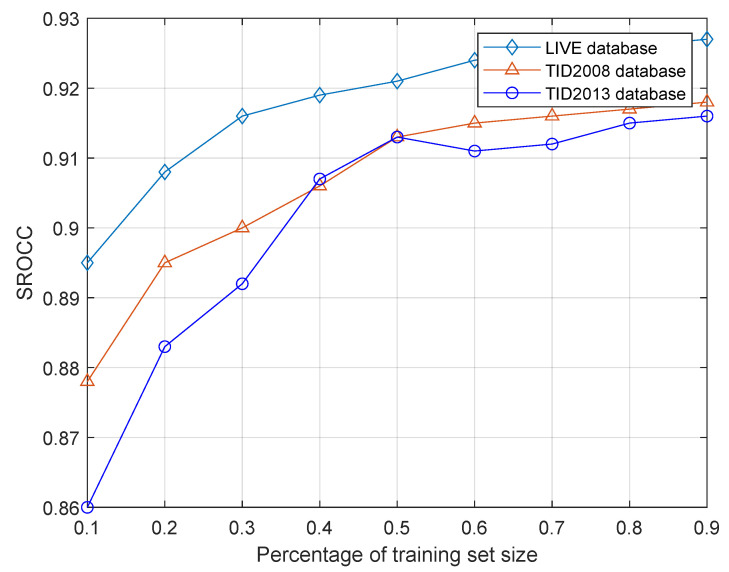
Results of SROCC under different training set size.

**Figure 9:**
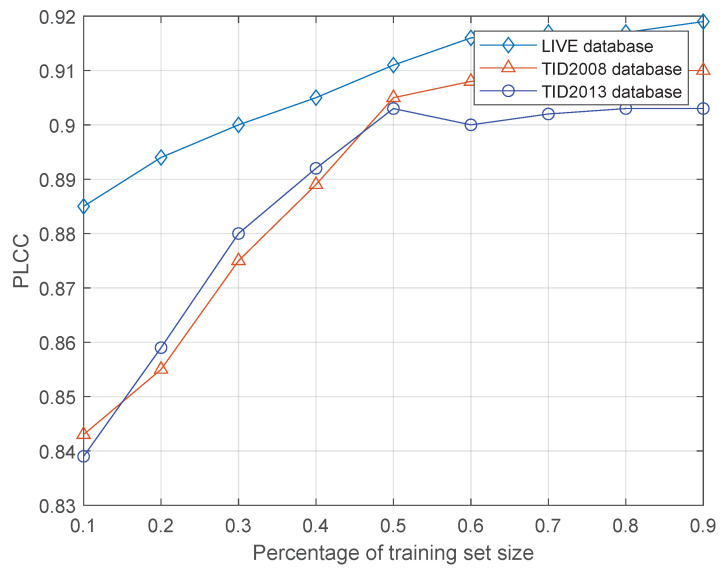
Results of PLCC under different training set size.

To verify the prediction performance of the network under different training set sizes, SROCC and PLCC are used as the functions of the training set size ratio, i.e., the training set sizes are 10% to 90%, respectively, and the training set is used to train the network. The data samples are divided into two different samples, 80%of which are used for training and the remaining 20% are for testing. Figure 8 and Figure 9 show the experimental results. Although the results of the network prediction decreased, they have not deteriorated severely as the size of the training set decreases. As shown in Figure 8, the percentage of SROCC performance in the training set is reduced from 90% to 30%, and the performance degradation is less than 5% in all three databases. However, in Figure 9, the performance of PLCC is slightly degraded, and the decreasing proportion is approximately 7%. The reason is that the designed network is a data-driven method, and the reduction of data volume affects the generalization of the network model to a certain extent. Furthermore, increasing the diversity of training data and the amount of data will help improve the adaptability of the network model to new samples.

#### 4.4.4. Computational Cost

In this section, we show the computational time of the proposed wavelet CNN model with different image data set. Please note that all the results are performed on a PC with 1.8 GHz CPU, and the memory is 16 GB, and the software tool is MATLAB R2018.we measure the processing time on three image data sets with 32 × 32 block size. Table 9 shows the average processing time. “LIVE + TID2008” in Table 9 refers to that the model is trained with LIVE database, and then tested with TID2008 data. “LIVE + TID2013” has the same meaning as “LIVE + TID2008”. As can be seen from Table 9, the training time of the proposed wavelet CNN model is relatively long, which is mainly because the proposed model runs in the CPU environment. To improve the calculation efficiency of the model, we will run it in GPU environment in the next work.

## 5. Conclusion

In this study, the idea of wavelet transform is introduced into the image quality evaluation based on CNN, and an improved nonreference quality evaluation method based on WCNN is proposed. This method uses the multiresolution characteristics of wavelet transform to acquire the sub-band images in multiple directions of the image as the input data of the CNN, thereby further increasing the diversity of the training data. Considering the sparse approximation capability of the wavelet transform to the image, the global and local information of the image is used to improve the effectiveness of image quality evaluation. On the contrary, the rich structural information of image information entropy can be used to reflect the degree of image change. In this study, information entropy is used as the weight of visual quality evaluation, which helps the predicted value of image quality to be close to human visual perception process. Finally, the results of experimental simulation and performance analysis proved the effectiveness and robustness of the proposed algorithm. As a result, the proposed method achieved 0.964 for SROCC,0.967 for PLCC, outperforming most of the existing NR-IQA methods in this article.

In the future, we will improve the existing network structure, and combine other methods, such as multi task learning method, to design a new image quality evaluation method.

## Figures and Tables

**Table 1:** PLCC comparison results of different methods on LIVE Database.

Methods	JP2K	JPEG	WN	Blur	FF	ALL
SSIM [4]	0.941	0.946	0.981	0.900	0.951	0.907
DIIVINE [10]	0.923	0.935	0.987	0.937	0.891	0.923
BRISQUE [11]	0.923	0.973	0.985	0.951	0.903	0.942
BLIINDS-II [12]	0.939	0.943	0.964	0.899	0.879	0.916
CNN [15]	0.953	0.981	0.984	0.953	0.933	0.953
BIECON [16]	0.965	0.986	0.970	0.945	0.931	0.961
Proposed	0.964	0.978	0.986	0.965	0.943	0.967

**Table 2:** SROCC comparison results of different methods on LIVE Database.

Methods	JP2K	JPEG	WN	b	FF	ALL
SSIM [4]	0.939	0.946	0.964	0.905	0.946	0.913
DIIVINE [10]	0.912	0.921	0.982	0.937	0.869	0.925
BRISQUE [11]	0.914	0.965	0.977	0.951	0.877	0.939
BLIINDS-II [12]	0.932	0.933	0.946	0.891	0.852	0.912
CNN [15]	0.952	0.977	0.978	0.962	0.908	0.956
BIECON [16]	0.952	0.974	0.980	0.956	0.923	0.961
Proposed	0.971	0.975	0.985	0.967	0.950	0.964

**Table 3:** Standard variance values of PLCC and SROCC on LIVE Database.

Distortions	JP2K	JPEG	WN	Blur	FF	ALL
PLCC	0.0045	0.0051	0.0057	0.0061	0.0049	0.0053
SROCC	0.0062	0.0063	0.0052	0.0062	0.0057	0.0059

**Table 4:** PLCC comparison results of different methods on TID2008 Database.

Methods	JP2K	JPEG	WN	Blur	ALL
SSIM [4]	0.955	0.931	0.826	0.929	0.894
DIIVINE [10]	0.931	0.878	0.843	0.870	0.886
BRISQUE [11]	0.839	0.932	0.836	0.881	0.890
BLIINDS-II [12]	0.923	0.867	0.834	0.869	0.881
CNN [15]	0.871	0.940	0.863	0.894	0.912
BIECON [16]	0.891	0.953	0.836	0.897	0.924
Proposed	0.885	0.942	0.903	0.913	0.927

**Table 5:** SROCC comparison results of different methods on TID2008 Database.

Methods	JP2K	JPEG	WN	Blur	ALL
SSIM [4]	0.963	0.935	0.817	0.960	0.902
DIIVINE [10]	0.924	0.866	0.851	0.862	0.889
BRISQUE [11]	0.832	0.924	0.829	0.881	0.896
BLIINDS-II [12]	0.917	0.859	0.815	0.857	0.873
CNN [15]	0.869	0.938	0.857	0.906	0.920
BIECON [16]	0.878	0.941	0.842	0.913	0.923
Proposed	0.890	0.934	0.905	0.917	0.926

**Table 6:** PLCC comparison results of different methods on TID2013 Database.

Methods	JP2K	JPEG	WN	Blur	ALL
SSIM [4]	0.925	0.843	0.796	0.824	0.859
DIIVINE [10]	0.901	0.696	0.882	0.860	0.794
BRISQUE [11]	0.919	0.950	0.886	0.884	0.900
BLIINDS-II [12]	0.893	0.683	0.872	0.864	0.791
CNN [15]	0.862	0.952	0.897	0.891	0.885
BIECON [16]	0.877	0.961	0.863	0.900	0.906
dipIQ [25]	0.948	0.973	0.906	0.928	0.894
CORNIA [32]	0.928	0.960	0.778	0.934	0.904
ILNIQE [33]	0.929	0.944	0.899	0.816	0.890
Proposed	0.894	0.957	0.912	0.898	0.915

**Table 7:** SROCC comparison results of different methods on TID2013 Database.

Methods	JP2K	JPEG	WN	Blur	ALL
SSIM [4]	0.925	0.846	0.785	0.833	0.862
DIIVINE [10]	0.857	0.680	0.879	0.859	0.795
BRISQUE [11]	0.906	0.894	0.889	0.886	0.883
BLIINDS-II [12]	0.914	0.888	0.695	0.857	0.839
CNN [15]	0.860	0.947	0.890	0.881	0.889
BIECON [16]	0.865	0.952	0.873	0.910	0.905
dipIQ [25]	0.926	0.932	0.905	0.922	0.877
CORNIA [32]	0.907	0.912	0.798	0.934	0.893
ILNIQE [33]	0.912	0.873	0.890	0.815	0.881
Proposed	0.902	0.949	0.911	0.906	0.913

**Table 8:** Performance results with different convolution kernel size.

Criteria	Convolution Kernel Size			
	3×3	5×5	7×7	9×9
PLCC	0.943	0.946	0.951	0.932
SROCC	0.947	0.943	0.950	0.939

**Table 9:** Computational time of the proposed model with different dataset (unit: min).

Image Dataset	LIVE	TID2008	TID2013	LIVE + TID2008	LIVE + TID2013
Time	13.82	14.53	15.74	14.61	15.30
